# The history of Teledermatology and its evolution, practical aspects, and prospects in Brazil^؆^

**DOI:** 10.1016/j.abd.2025.501208

**Published:** 2025-10-27

**Authors:** Kevin Yun Kim, Lung Wen Chao, Cyro Festa Neto

**Affiliations:** a Department of Dermatology, Universidade de São Paulo, São Paulo, SP, Brazil; b Department of Pathology, Universidade de São Paulo, São Paulo, SP, Brazil

**Keywords:** Dermatology, Teledermatology, Telemedicine

## Abstract

Teledermatology services in Brazil have a rich history, with several ongoing projects boosting academic research and providing efficiency gains by reducing waste, humanizing healthcare, and ensuring greater quality and speed in patient assistance. Reviewing the fundamentals and history of Teledermatology, along with the continuing evolution of Telemedicine services in Brazil and its practical aspects and future prospects, might bring better comprehension of how to further develop and improve these services.

## Introduction

### Fundamentals and history of Teledermatology

Although strongly associated with technological innovations in the areas of information technology and telecommunications in recent decades, especially the Internet, the history of medicine with remote support actually dates back to probably even before the Middle Ages.^1^ In ancient times, for example, sending letters via ships or horse-mounted messengers containing clinical information, hand-drawn pictures, and even urine samples to doctors or healers requesting a report with expert opinion could already be considered a rudimentary form of health services provided remotely.

The authors can take as a reference that the use of technologies in the provision of health services probably began with the invention of the stethoscope by the French physician René-Théophyle-Hyacinthe Laennec in 1819. Although it still required the physical presence of the physician, for the first time in History, it was possible to make clinical and diagnostic inferences without the need for direct contact with the patient during physical examination.^2^

The incorporation of new technologies for the provision of remote health services over time occurred with the advent of increasingly sophisticated forms of communication: starting in the 19^th^ century with the expansion of mail services, moving on to the creation and large-scale implementation of the telegraph, and later telephone, radio and closed-circuit television networks, culminating in the global computer network – the Internet, created in 1969 – providing a high-speed, worldwide connection experience never seen before.^3^

The modern term Telemedicine has been associated with the idea of using electronic technologies (devices and telecommunications) to provide medical services remotely, having gained important meaning because of the war efforts during the Cold War and the Space Race, a time when many pioneering technological advances were achieved by government and military agencies and were later incorporated into the civil and health sectors.^4^ With all the innovation brought about by computerization and the development of high-speed and stable Internet connections, written and visual information began to circulate globally in practically real time.

In the field of Dermatology, it is very difficult to separate telemedicine from the significant advances in digital images (photographs and videos), especially since the last decade of the 20^th^ century, which have made it possible to generate visual information with ease and increasingly higher resolutions at progressively lower costs.

Although controversial, the invention of photography is often attributed to the Frenchman Joseph Nicéphore Niépce, who in 1826 managed to obtain the oldest permanent photograph still in existence,^5^ leading mankind towards a new era as for the first time there was a shift from artistic drawing to realistic or real and reproducible documentation with the same characteristics under the same conditions. The process has been perfected ever since, but it took more than a century until the first digital processing of a photograph was carried out by the US government space agency, the National Aeronautical and Space Administration (NASA), in the mid-1960s, when cameras and transmitters were attached to a lunar probe in order to transmit analog video/image signals.^6^ Technological advances were gradually implemented in cameras, leading to the creation of the first digital camera prototype by Steven Sasson in 1975, at the time a young engineer working for Kodak. Despite being innovative, Sasson's invention was not met with enthusiasm by Kodak's own executives, which eventually led to the company's bankruptcy in 2012.^7^

It was only in the early 1980s that these technologies became more popular, with the initial milestone being the commercialization of the Mavica analog models produced by Sony in 1981. The first professional digital camera was released by Kodak in 1991, allowing images with up to 1.3 megapixels of resolution. However, the extremely high prices until the early 1990s made widespread access to these machines prohibitive. The revolution in popular access to digital cameras occurred only approximately 30-years ago, in mid-1995, when Apple's QuickTake 100 and Kodak's DC 40 models were launched at prices below the US$1,000.00 barrier.^8^

**Figure 1:**
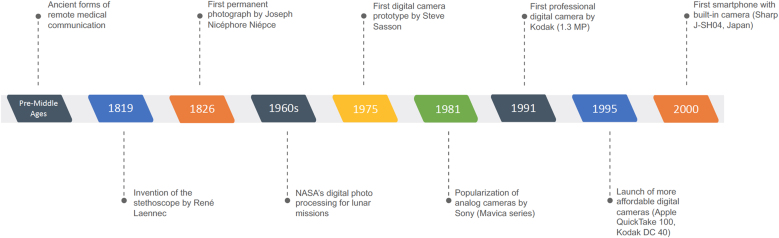
Evolution of remote medical communication and imaging until the smartphone era.

Closely following the evolution of digital cameras, smartphones (cell phones with advanced features and personal computer resources) have also shown rapid development in terms of processing capacity and production/capture of high-resolution images. The first smartphone with a built-in digital camera was the J-SH04 model produced by Sharp and launched in Japan in November 2000.^9^ Since then, new models equipped with more powerful digital cameras and extra versatile features are released at competitive prices yearly. The development of digital cameras attached to smartphones was extremely important for Telemedicine since images with higher resolution, combined with appropriate Digital Photography techniques, allow for a greater level of detail, making them suitable for clinical assessment (Fig. 1).^10^

According to data obtained by the 28^th^ Annual Survey on the Use of Information Technology carried out by the Getúlio Vargas Foundation (FGV), the estimated number of smartphones in Brazil at the end of 2017 was 208 million devices, which means 1 smartphone per inhabitant in the country.^11^ Easy access to mobile devices with increasingly greater processing and image generation power has led to a significant increase in the use of these devices in the digital world, cloud data processing and social networks. At the same time, in the medical field, there has been an increase in application tests on several areas, including Ophthalmology,^12^ Dermatology,^13^, ^14^ Plastic Surgery,^15^ Endocrinology,^16^ Neurosurgery^17^ and Radiology,^18^ being applied in a versatile way as a means of remote screening, diagnosis, prevention and post-operative follow-up. Images of clinical cases are often shared on messaging apps or social networks such as WhatsApp® (Meta Platforms Inc, Menlo Park, California, USA) for discussion with other medical colleagues^19^ or through smartphone apps created especially for this purpose.

Due to the widespread, often informal, use of smartphones as everyday tools and sometimes in applications that could be associated with Telemedicine, some fundamental aspects such as patient data privacy, legal support for doctors and healthcare institutions, data security and storage, and medical education in Telemedicine and digital photography techniques still need to be better discussed and consolidated.^20^ As smartphones are portable devices that can easily be lost, compromised, or stolen, healthcare professionals need to become increasingly aware of the responsibility of carrying patients' private and sensitive data on their personal devices.

Dermatology may be a medical specialty prone to the use of Telemedicine because of its large visual component, and a growing application of Teledermatology has been noted on a global scale over the years.^21^ Multiple studies have corroborated the clinical effectiveness,^22^, ^23^ cost-benefit,^24^, ^25^ high satisfaction rates^21^, ^26^ and reduced waiting times for specialized dermatological evaluation in health systems^23^, ^27^ through the incorporation of Teledermatology, showing potential applicability with high rates of sensitivity and diagnostic agreement in relation to the traditional clinical examination in several skin pathologies in both outpatient and hospital settings.^13^, ^26^, ^28^ Due to the high prevalence of skin cancers and their significant economic impact on public and private health systems in several countries,^29^, ^30^ Cutaneous Oncology in particular has been the focus of much of the Teledermatology studies.^14^, ^22^, ^27^, ^31^, ^32^, ^33^, ^34^

### The evolution of Telemedicine in Brazil

Telemedicine has been regulated in Brazil by the Federal Council of Medicine (CFM) since 2002, through Resolution 1643/2002,^35^ which already provided for the teleinterconsultation modality. In 2011, the Brazilian Ministry of Health proposed the use of Telemedicine resources to solve problems in the Primary Care level of its public Unified Health System (SUS), through the publication of Ordinance nº 2,546 of October 27, 2011,^36^ which defined the concept of Second Formative Opinion, a source of information originated through teleconsultations that deal with issues relevant to the SUS and with the possibility of answering questions and needs of other health workers, aiming to increase the resolution capacity in similar cases or situations.

Since 2020, the Brazilian Ministry of Health has established the National Health Data Network (RNDS) through Ordinance nº 1,434 of May 28, 2020,^37^ a strategic program targeting digital transformation of healthcare in the country aiming to promote interoperability to enable the exchange of information and data sharing between the different sites within the Health Care Network, allowing the transition and continuity of care in the public and private sectors.

Brazilian legislation regarding Telemedicine progressed significantly during the COVID-19 pandemic, culminating in Resolution nº 2314/2022^38^ of the CFM, which definitively regulates Telemedicine, defining this practice as a medical act, as a form of medical services mediated by communication technologies, defining clearly seven different telemedicine modalities (Table 1).

**Table 1:** Telemedicine modalities and Telehealth definitions established in Brazil.

	Definition
**Teleconsultation**	A non-face-to-face medical consultation, mediated by Digital, Information and Communication Technologies (DICTs), with the doctor and patient located in different spaces.
	
**Teleinterconsultation**	Exchange of information and opinions between physicians, with the help of DICTs, with or without the presence of the patient, for diagnostic or therapeutic, clinical or surgical assistance.
	
**Telediagnosis**	The medical act at a distance, both geographically and/or temporally, with the transmission of graphs, images and data for issuing a report or opinion by a qualified specialist physician in the area related to the procedure, in response to the request of the attending doctor.
	
**Telesurgery**	Surgical procedures are performed remotely, using robotic equipment and mediated by safe interactive technologies.
	
**Telemonitoring or medical televigilance**	The act performed under the coordination, indication, guidance and supervision of a doctor for remote monitoring or surveillance of health and/or disease parameters, through clinical evaluation and/or direct acquisition of images, signals and data from equipment and/or devices attached or implanted in patients at home, in specialized chemical dependency clinic, in a long-term care facility for the elderly, in a clinical or home hospitalization regime or in the transfer of patients until their arrival at a health establishment.
	
**Teletriage**	The act performed by a physician with evaluation of the patient's symptoms, remotely, for outpatient or hospital regulation, with definition and direction of the patient to the appropriate type of assistance needed or to a specialist.
	
**Teleconsulting**	An act of consultancy mediated by DICTs between physicians, managers and other professionals, with the purpose of providing clarifications on administrative procedures and health actions.
	
**Telehealth**	Method of providing health services remotely, through the use of information and communication technologies, which involves, among others, the secure transmission of health data and information, through texts, sounds, images or other appropriate forms.

In the same year, Ordinance Office of the Minister/Ministry of Health nº 1,348 of June 2, 2022^39^ was published, which provides for Telehealth actions and services within the scope of the SUS, with the objective of regulating and operationalizing the use of information and communication technologies in remote assistance, education, research, prevention of diseases and injuries, management and promotion of citizen health, and also Law nº 14,510 of December 27, 2022,^40^ which authorizes and regulates the practice of Telehealth throughout the national territory.

Due to the restrictions on mobility caused by the pandemic, the Brazilian Society of Dermatology (SBD), aiming at the safe practice of remote evaluations that had increased dramatically, published in 2021 the first version of the Manual of Good Practices in Teledermatology, in addition to organizing a series of webinars on the subject, seeking training and continuing medical education of specialists. This manual had a second version published in 2022^41^ containing an update in accordance with CFM resolution nº 2314/2022.^38^

Therefore, despite being fully authorized and regulated only recently, Telemedicine and, by extension, Teledermatology, have been a widely debated topic in Brazilian society due to its high relevance and potential impact, advantages highlighted even more in the context of social isolation imposed during the COVID-19 pandemic.

## Methods

A literature review search strategy was performed on the PubMed® platform, maintained by the National Library of Medicine (Bethesda, Maryland, USA) and which gathers records from the MEDLINE database, using the terms “Dermatology” AND “Brazil”. All abstracts were analyzed, and only unique published articles reporting on Teledermatology projects developed and executed in Brazil were included in the analysis after reading of the full-text articles and confirmation of pertinence.

## Results

**Figure 2:**
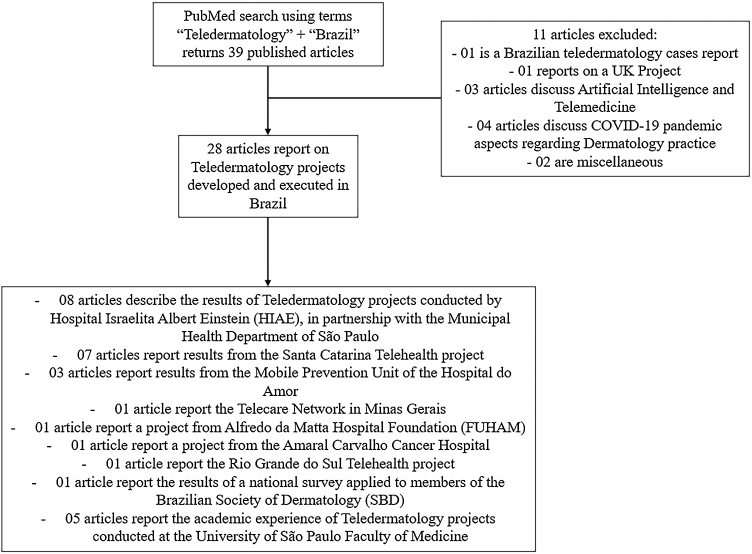
Summary of the PubMed research results regarding Teledermatology studies in Brazil.

The research strategy returned 39 original published and indexed scientific articles, of which 28 report on Teledermatology projects developed and executed in Brazil (Fig. 2).

Of these, 08 articles describe the results of Teledermatology projects conducted by Hospital Israelita Albert Einstein (HIAE)^42^, ^43^, ^44^, ^45^, ^46^, ^47^, ^48^, ^49^ in partnership with the Municipal Health Department of São Paulo. In July 2017, the city of São Paulo, through its Municipal Health Department, agreed in a partnership with the Telemedicine service of the HIAE with the aim of significantly reducing the waiting list for dermatological care, where dermatologists from HIAE would remotely analyze the needs of patients based on clinical information and photographic images of skin lesions collected by nurses from SUS health units in the capital of São Paulo ‒ if any additional diagnostic or surgical procedure was necessary, HIAE was responsible for providing a referral network and its own infrastructure with mobile units to meet the demand.^50^

Other published studies present results from projects where primary care professionals benefit from specialized remote assessment through partnerships with university and/or reference services: from the Santa Catarina Telehealth project, the largest Teledermatology initiative in Brazil detailed further in this article, there are 7 articles published;^51^, ^52^, ^53^, ^54^, ^55^, ^56^, ^57^ from the Mobile Prevention Unit of the Hospital do Amor, in the city of Barretos, 3 articles;^58^, ^59^, ^60^ from the Telecare Network in Minas Gerais, 1 article;^61^ from the Alfredo da Matta Hospital Foundation (FUHAM) in Amazonas, 1 article;^62^ from the Amaral Carvalho Cancer Hospital, in the city of Jaú, 1 article;^63^ and from the Rio Grande do Sul Telehealth project, 1 article.^64^ The results of a national survey applied to members of the Brazilian Society of Dermatology (SBD) were also published,^65^ a study led by a research team from the State University of Campinas (UNICAMP). However, despite these initiatives, there are still a lot of opportunities for expansion of Teledermatology in Brazil, since according to information available on the Brazilian Open Data Portal^66^ there are currently around 38,000 Primary Care Units operating in Brazilian territory.

Many studies have already addressed the pedagogical importance of Teledermatology for medical education in academic environments.^67^, ^68^ At the University of São Paulo Faculty of Medicine (FMUSP), the institution where the remaining 5 published articles^13^, ^28^, ^69^, ^70^, ^71^ were developed, some academic pilot projects involving Tele-education for non-medical workers have already been developed with promising results - online training platforms for the prevention of venereal diseases targeting beauty salon employees^70^ and a Distance Education (EAD) system for the training and education of non-medical health professionals in screening of skin lesions with malignant potential,^71^ in addition to a website offering organized content for training in early recognition of melanoma by first-year medical students.^31^ Two other studies, involving physician participants, studied the application of asynchronous Teledermatology: one evaluated its role in supporting clinical diagnosis of leprosy performed by primary care physicians, with promising and low-cost results;^28^ the other assessed its application through smartphones to discuss cases of patients admitted to a reference hospital and evaluated by Dermatology residents.^13^

In this same institution, in 2001, an Internet-based dermatological consultation system (cyberambulatory) called “Telederma” was developed, where dermatology residents registered patients’ records containing clinical data and digital photographs of skin lesions so that they could later be evaluated by more experienced dermatologists. In order to maintain the academic aspect of this virtual outpatient clinic, fundamentals of Problem-Based Learning and Evidence-Based Medicine were incorporated through an electronic tutor (cybertutor), with the online provision of diagnostic guidelines, bibliographic references, a database on drug interactions, didactic classes taught by FMUSP professors, and performance evaluation methods of participating physicians.^69^, ^72^ In mid-2003, this same cyberambulatory would be used in the training of residents in Sanitary Dermatology in the specialized service for Sexually Transmitted Infections (STI) of the Geraldo de Paula Souza School Health Center, led at the time by Prof. Dr. Luiz Jorge Fagundes.

Regarding analysis of the factors involved in the use of photographs for dermatological diagnosis, a study conducted at FMUSP made it possible to identify that the systematization and implementation of dermatological tele-propaedeutics should be analyzed under a set of classifications of the types of skin lesions and their clinical relevance, forming a matrix, demonstrating that in several situations additional clinical information is necessary. It concluded that skin diseases with clinically atypical or moderately typical lesions and with palpable morphology (e.g., infiltrated lesions, subcutaneous tumors) are more difficult to diagnose with the aid of digital photography alone, with clinical data and additional information being of fundamental importance to increase diagnostic accuracy. Furthermore, the systematic implementation of Teledermatology allowed improvement in remote diagnostic performance comparable to in-person assessment.^73^

## Discussion

### Practical aspects

A great example of how to bring Teledermatology to practice, integrated with existing health systems, is the main and largest Brazilian initiative with the incorporation of Teledermatology into the regulatory process of the SUS in the state of Santa Catarina, which has been in non-stop operation since October 2013. In a continental-sized country like Brazil, with several economic disparities within its national territory and a large variance regarding access to quality healthcare within different regions of the country, if most of the Primary Care Units are integrated in a nation-wide Teledermatology project such as the one running in Santa Catarina, the impact on healthcare delivery and cost-efficiency could be huge.

The use of Teledermatology in the state of Santa Catarina, located on the Southern region of Brazil, was formalized with Resolution 366/CIB/13 from August 22, 2013,^74^ issued by the State Bipartite Health Management Committee (CIB/SC), which approved the use of Telediagnosis in Dermatology for risk assessment and regulation of patient flow originating from Primary Care services within the medical specialty of Dermatology. Consequently, ever since, in Santa Catarina, patient triage and referrals via the Brazilian National SISREG System began officially to be conducted through Telemedicine.

The platform was developed by the Federal University of Santa Catarina (UFSC) and is used as a tool for both triaging patients with skin diseases and for risk assessment by the State Health Department of Santa Catarina (SES-SC). The Teledermatology reports issued through the platform are part of the decision-making process for Primary Care physicians and support the regulatory physician in referring patients to specialized services.

This service is well-established, with over 300,000 Teledermatology examination reports conducted until 2024, and is currently present in 15 other federative units through a national program.^75^, ^76^ Furthermore, the implementation of the Teledermatology tool has been able to reduce both direct and indirect costs to the Brazilian Public Healthcare System through a decentralized infrastructure for patient triage with access to risk classification, aiding in the appropriate referral of patients, reducing travel expenses, queues, wait times, and also eliminating unnecessary intermediary appointments.^77^

In the USA, by 2017, among the 40 nationally active teledermatology programs, 70% provided direct-to-patient care, and 72% used store-and-forward models only.^78^ As for the United Kingdom National Health Service (NHS), since October 2018, all General Practitioners in England are required to use the NHS e-Referral Service (e-RS), a mostly asynchronous physician-to-physician platform, to refer to consultant-led new outpatient appointments.^79^ Despite global changes prompted by the COVID-19 pandemic caused synchronous models (primarily video visits) to supplant asynchronous models (store-and-forward or shared digital photographs) as the predominant modality of teledermatology care in various locations,^80^ in the published Brazilian teledermatology projects asynchronous models still largely prevail, possibly due to the synchronous models technological barriers, as they require greater infrastructure to achieve optimal video quality and accessibility.

Yearly, Internet access in Brazil is increasing rapidly, especially in rural areas, where homes with Internet access increased from 35.0% to 81.0% between 2016 and 2023,^81^ which might also increase access to teledermatology services in remote areas. This is specially benefitial considering that the number of Brazilian medical school graduates is rapidly increasing – from 2020 to 2022, graduated physicians increased by 8.6%, an over five-fold difference compared to the population growth on the same period of only 1.6% ‒ but the number of Dermatology residents decreased by 30.9% between 2018 and 2021,^82^ leading to a proportional lack of specialists – therefore, teledermatology has a great potential to increase access to specialized opinion to non-specialist physicians, in order to improve proper healthcare access.

### Future prospects

Exciting future prospects regarding Teledermatology which still requires further studies, but in fields where Brazilian institutions definitely have the potential and opportunity to develop, include application of Teledermatology services with an academic approach involving medical residents and the determinant factors that impact telemedicine usage and training among physicians in-training; integration of Telemedicine within smart homes and its potential impacts on the general population and Lifestyle Medicine;^83^ and the integration of Artificial Intelligence (AI) and Teledermatology, which has already been showing promising signs on improving risk assessment, diagnostic accuracy and quality control, but still present some gaps such as proper regulation and standardization, development of position statements and guidelines, and also clarification on civil and ethical aspects of its usage.^84^, ^85^ Rapidly advancing projects such as DermAI keep continuously developing novel deep learning techniques to improve automated classification of skin lesions.^86^

Although AI-powered Teledermatology widespread populational initiatives still lack, Edge Health, a specialist UK healthcare analytics consultancy, was commissioned by Health Innovation East Midlands (one of 15 organizations across England acting together as the innovation arm of the NHS) to carry out an independent evaluation of the effectiveness of a pilot tested across University Hospitals of Leicester (UHL) sites starting from March 2022.^87^ This collaborative project was designed to respond to the local need for improved patient access to dermatology diagnostics, notably skin cancer, using a Skin Analytics AI-powered Teledermatology solution called DERM. Their preliminary October 2023 report confirmed that the pilot led to a reduction in referrals, freeing up 1450 outpatient appointments, and there was also a clinical time saving of 263 minutes per 100 patients, with a cost-benefit ratio of 1.05 (for each pound invested in the AI-powered solution, the health system received £1.05 in benefits).

## Conclusion

In view of these data and the vast potential for expansion of Teledermatology services in Brazil, especially Teleinterconsultation assisted with AI, which has strategic potential for streamlining processes and efficiency gain not only from an economic point of view, but also as a way of humanizing care by reducing waste and ensuring greater quality and speed in assistance, it is extremely important that Teledermatology keep advancing to provide better patient experience and training for future professionals, including dermatologists and other physicians and health care workers.

## Financial support

None declared.

## Authors’ contributions

Kevin Yun Kim: Conceptualization; data curation and analysis; writing original draft; critical review of literature; final approval of the manuscript.

Chao Lung Wen: Conceptualization; critical review of important intellectual content; effective supervision and research orientation; critical review of literature; final approval of the manuscript.

Cyro Festa Neto: Conceptualization; critical review of important intellectual content; effective supervision and research orientation; critical review of literature; final approval of the manuscript.

## Research data availability

Does not apply.

## Conflicts of interest

None declared.
